# Probing Hepatic Glucose Metabolism via ^13^C NMR Spectroscopy in Perfused Livers—Applications to Drug Development

**DOI:** 10.3390/metabo11110712

**Published:** 2021-10-20

**Authors:** Corin O. Miller, Jin Cao

**Affiliations:** Department of Translational Imaging, Merck & Co., Inc., Kenilworth, NJ 07033, USA; jin.cao@merck.com

**Keywords:** perfused liver, nuclear magnetic resonance spectroscopy, NMR, MRS, stable isotope, ^13^C (carbon-13), glucose metabolism, glycogen metabolism, gluconeogenesis, lipid metabolism, de novo lipogenesis, fatty acid oxidation

## Abstract

Despite being first published over 40 years ago, the combination of ^13^C nuclear magnetic resonance spectroscopy (NMR) and the isolated perfused liver preparation remains a unique and relevant approach in investigating the effects of pharmacological interventions on hepatic metabolism. The use of intact, perfused livers maintains many metabolic reactions at their respective rates in vivo, while the use of ^13^C-labelled substrates in combination with ^13^C NMR allows for a detailed study of specific pathways, as well as the design of robust assays which can be used to evaluate novel pharmacological agents. In this review article, we share some of the methods used to probe glucose metabolism, and highlight key findings and successes derived from the application of this specialized technique to the area of drug development for diabetes and related metabolic disorders.

## 1. Introduction

Throughout the recent decades, there have been numerous novel therapeutic agents approved for the treatment of diabetes and related metabolic disorders. Consistent with the heterogenous nature of the etiology of diabetes, these treatments elicit their therapeutic effects via modulation of different metabolic pathways such as insulin sensitivity [1], renal glucose reabsorption [2], glucose-dependent insulin secretion [3], and hepatic glucose production [4]. Consistent with the multiple mechanisms of action (MOA) and target organs related to different diabetes therapies, there also are many different pre-clinical models of diabetes and related metabolic disorders. These range from high fat and/or sugar diet-induced models [5,6], models of leptin dysfunction [7], polygenic models [8], and pharmacologically induced models [9].

Due to the numerous different therapeutic MOAs and diabetic animal models, selection and validation of both novel drug targets and pre-clinical in vivo models for diabetes can be a daunting task. A useful step in this process is to evaluate the effects of novel compounds on specific metabolic pathways in a simpler ex-vivo system. For liver-targeted therapies, the isolated, perfused liver represents a very useful platform to assess the effects of novel compounds in the absence of external inputs. Another ubiquitous approach in the study of metabolic pathways is the use of nuclear magnetic resonance (NMR) spectroscopy with ^13^C-labelled stable isotopes. Despite having an inherently low NMR signal due to its low gyromagnetic ratio and natural abundance of 1.1%, ^13^C NMR can still be used to detect the incorporation of ^13^C-labelled substrates into certain highly abundant end products of metabolic pathways (e.g., glycogen, triglycerides). Additionally, with appropriate hardware ^13^C NMR-based approaches with stable isotopes can often be translated into preclinical species, such as rodents and non-human primates, as well as humans, allowing for the potential of using similar NMR-based readouts from ex-vivo systems through the clinic. In this article, we review some of the successes where the perfused liver approach with ^13^C NMR was used to develop assays to evaluate novel pharmacologic compounds or elucidate the therapeutic MOA of novel targets. Where allowed by intellectual property guidelines, we describe the characterization of specific compounds. Where not possible, we describe the characterization of commercial tool compounds, or endogenous ligands. We focus this review on drug development related to glucose metabolism as applied to diabetes. 

## 2. Perfused Liver Methods

**Figure 1:**
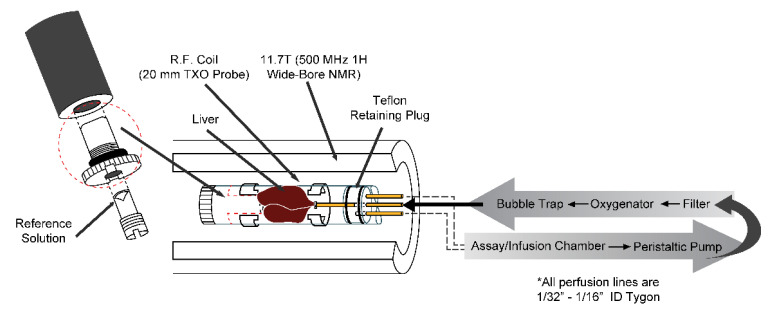
Schematic of the perfused liver apparatus. The liver sits inside a custom-made 20-mm NMR tube which is inserted into a 20-mm ^13^C/^31^P/^1^H TXO probe. The observed ^13^C NMR signals arise from the liver and approximately 10 mL of the surrounding perfusate. The assay/infusion chamber allows for periodic sampling of the perfusate and administration of exogenous compounds. The oxygenator consists of Silastic tubing continually bathed in 95% O_2_/5% CO_2_ gas and wound around a hollow cylinder filled with water, the temperature of which is controlled via external water bath. *All other perfusion lines are Tygon tubing of inner diameter 1/32” to 1/16”. Reprinted from reference [11].

^13^C-labelled substrates used in these studies were purchased from Sigma-Aldrich (St. Louis, MO, USA) and were of isotopic enrichment > 99%. Animals were studied under the purview of Institutional Animal Care and Use Committee (IACUC) of Merck & Co., Inc., Kenilworth, NJ, USA, and all applicable regulations and laws pertaining to the use of laboratory animals were followed. A schematic of the perfused liver approach is shown in Figure 1. A detailed description of the perfused liver technique can be found elsewhere [10]. Briefly, mice (various strains and ages) were anesthetized (Pentobarbital IP, 100 mg/kg), the portal vein was cannulated and tied off, and the liver was excised and perfused with a pre-oxygenated Krebs bicarbonate-buffered solution. The liver was then placed into a 20-mm NMR tube and the combined assembly was inserted into a 11.7-T (500 MHz) wide bore NMR spectrometer (Bruker Biospin, Billerica, MA, USA). ^31^P NMR spectra were initially obtained in each study to measure levels of ATP and inorganic phosphate (Pi) as an overall check of liver viability. For selected studies, a second ^31^P NMR spectrum was also acquired at the end of the study and used to verify the following: (1) <10% change in the intensity of the ATP resonances; and (2) a lack of upfield shift of the chemical shift of the Pi resonance (which would indicate a decrease in intracellular pH and poor liver viability). In most cases, a baseline (natural abundance) ^13^C NMR spectrum was acquired prior to administration of ^13^C-labelled substrates and was subtracted from all subsequent NMR spectra which were acquired serially for 60–120 min. The integrals of the NMR signals from the ^13^C-labelled metabolites of interest were then calculated by fitting the resonances to a Lorentzian line shape model using in-house developed curve fitting routines in Matlab (The Mathworks R2016a Inc., Natick, MA, USA). These integrals were converted to absolute units (μmoles) by comparison of integrals from ^13^C NMR spectra of standard solutions acquired under identical conditions, or, where applicable, by comparison of biochemical measurements of a particular metabolite in perfusate samples.

## 3. Glycogen Metabolism

### 3.1. Glycogen Metabolism from [2-^13^C] Pyruvate

In the perfused liver, synthesis of glycogen can be achieved from several substrates (e.g., pyruvate, fructose, glycerol, and glucose). The use of [2-^13^C] pyruvate is sometimes a convenient substrate for glycogen synthesis because its concentration in the perfusion buffer (aka perfusate) has little effect on the rate of glycogen synthesis, which will thus remain relatively constant as the pyruvate is consumed. This is in contrast to ^13^C-glucose where the concentration of glucose in perfusate directly influences glycogen synthesis [12]. Additionally, using a glycogenic substrate other than ^13^C-glucose allows the potential to monitor the breakdown of any newly formed ^13^C-glycogen to ^13^C-glucose, which would otherwise be obscured by the larger NMR signal of the ^13^C-glucose substrate itself. Finally, the choice of ^13^C label in position C2 of pyruvate is optimal because C1 is lost to CO_2_ in the pyruvate dehydrogenase (PDH) reaction, while C3 has an NMR resonance close to the methyl resonances from many other metabolic products of interest, and thus may complicate the spectra. 

**Figure 2:**
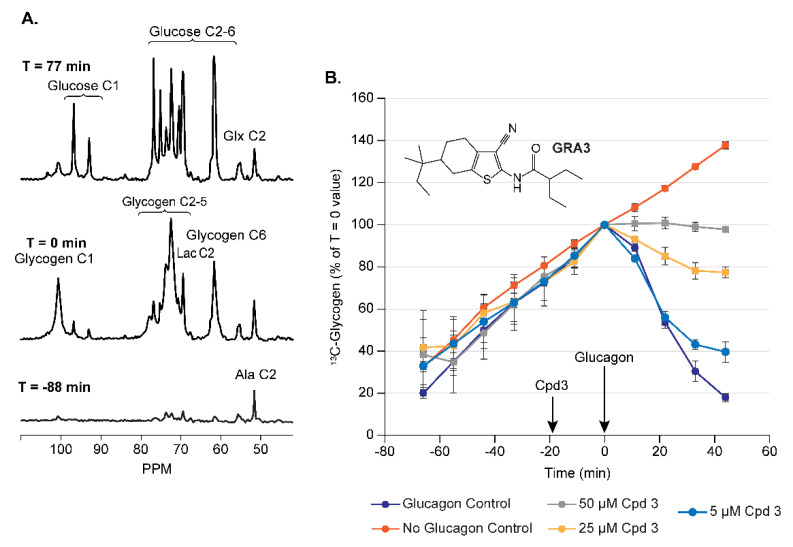
The development of a perfused liver assay for the assessment of glucagon receptor antagonists. (**A**) Sample ^13^C NMR spectra over time from mouse livers harvested during the dark cycle metabolizing [2-^13^C] pyruvate. There is an increase and decrease in the [1-^13^C] glycogen NMR signal before and after the addition of 50 pM of glucagon, respectively, at T = 0 min. (**B**) The dose-dependent modulation of this process by a novel small molecule GRA3. Acquisition parameters for panel A as well as all other spectra in this review are as follows: 30° pulse, TR = 560 ms, NS = 800 averages, broadband GARP ^1^H decoupling, 11 min/spectrum. Adapted from reference [13].

Shown in Figure 2 are spectra from a perfused liver presented with approximately 7 mM of [2-^13^C] pyruvate [13]. In addition to the appearance of the [1-^13^C] glycogen signal, other ^13^C-labelled metabolites can be observed. Many of these will form the basis for assays described later in this article. For studies of glycogen metabolism, mouse livers were harvested and studied in the middle of the dark cycle, which was found to favor glycogen synthesis from pyruvate over glucose production.

#### 3.1.1. Assay Development for Glucagon Receptor Antagonists (GRA)

Glucagon is the counter-regulatory hormone to insulin and promotes glycogen breakdown in the liver and the subsequent release of glucose into the circulation as part of the physiological control of plasma glucose. The inhibition of this process via GRAs thus represents a potential mechanism to lower circulating glucose levels in diabetics. Shown in Figure 2A are spectra from a perfused mouse liver presented with approximately 7 mM of [2-^13^C] pyruvate. As shown, the perfused liver can be used to monitor the synthesis of ^13^C-glycogen from [2-^13^C] pyruvate (bottom vs. middle spectra), and then the degradation of this newly formed ^13^C-glycogen from acute administration of 50 pM of glucagon to the liver (middle vs. top spectra). This process of glucagon-induced glycogen breakdown can then be modulated by the addition of novel GRA compounds prior to the addition of glucagon to the perfusate. Figure 2B shows the time courses of [1-^13^C] glycogen for various doses of GRA3, the chemical structure of which is shown [14]. As shown in these figures, the effect window (i.e., the difference in the response between glucagon and vehicle) is large compared to the standard deviations which makes for an excellent assay to evaluate novel GRA compounds. To further enhance the relevance of this assay to human biology, livers from mice expressing the humanized glucagon receptor (hGcgR) were used [15]. A dose titration of glucagon itself in this assay (data not shown) yielded an EC_50_ of approximately 20 pM (70 ng/mL) which is consistent with in vivo data [16], highlighting the fact that the perfused liver recapitulates normal physiology. 

#### 3.1.2. Assay Development for GLP1/Glucagon Dual Receptor Agonists

**Figure 3:**
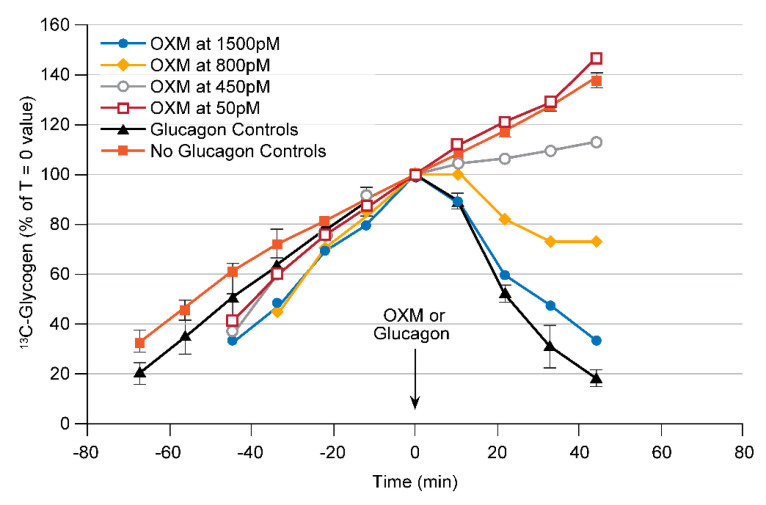
The development of a perfused liver assay for the assessment of glucagon receptor (GcgR) activation for application to GLP1R/GcgR dual agonists. ^13^C NMR spectra used in this assay were similar to those in Figure 2A. The dose titration of native oxyntomodulin is shown, with an approximate EC_50_ for induction of glycogenolysis of 700 pM in livers from mice expressing the humanized glucagon receptor. Adapted from reference [19].

The GLP1 receptor is a potent regulator of glucose homeostasis via enhancing pancreatic secretion of insulin in a glucose-dependent manner [3], and several current commercial diabetes therapies have successfully exploited this. In addition, there is evidence from studies of the endogenous peptide hormone Oxyntomodulin (OXM) that compounds which activate both the GLP1R and the GcgR simultaneously may confer an additional body weight lowering benefit [17]. Thus, dual agonists of the GLP1R/GcgR may yield an additional therapeutic benefit over GLP1R agonists alone. One of the key questions in the development of these compounds is the relative potency on each receptor. For this, two separate pharmacodynamic assays are required. The effect on the GLP1R can generally be determined via traditional measures of plasma glucose and insulin; however, there is no standard in vivo assay of GcgR activation that would work in the presence of simultaneous GLP1R activation. Since liver does not express the GLP1R [18], the perfused liver provides a unique paradigm to evaluate potential GLP1R/GcgR agonists for effects on the GcgR alone. Shown in Figure 3 are time courses from perfused hGcgR mouse livers similar to those shown in Figure 2B for the endogenous peptide OXM; however, the difference is that this assay can now be used to evaluate the effect on GcgR activation (via induction of glycogenolysis) independent from its effects on the GLP1R. 

### 3.2. Glycogen Metabolism from [1-^13^C] Glucose

**Figure 4:**
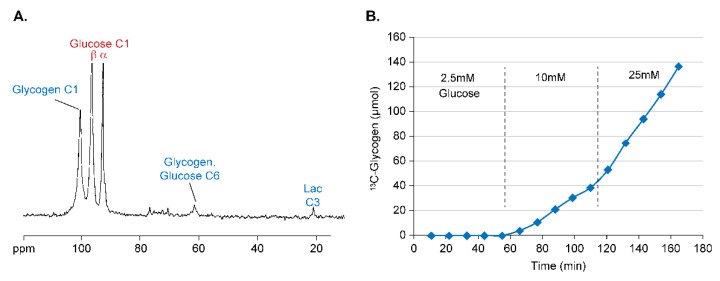
The measurement of ^13^C-glycogen synthesis from [1-^13^C] glucose. (**A**) ^13^C NMR spectrum from a perfused liver after 50 min of metabolizing 20 mM of [1-^13^C] glucose. The end products [1-^13^C] glycogen, [6-^13^C] glycogen, and [3-^13^C] lactate are observed. (**B**) The time course of [1-^13^C] + [6-^13^C] glycogen for a single liver presented with different perfusate concentrations of [1-^13^C] glucose. Reprinted from reference [11].

A more traditional substrate for hepatic glycogen synthesis is [1-^13^C] glucose, which is preferred over [U-^13^C] glucose in perfused liver studies due to its lack of ^13^C-^13^C couplings which results in simpler NMR spectra. In the liver, glucose is transported into the hepatocyte by GLUT2 and is subsequently phosphorylated and converted to glucose-6-phosphate (G6P) by glucokinase (hexokinase IV). The kinetics of glucokinase are different than most other hexokinases in that its rate of glucose phosphorylation varies over the normal physiological range of plasma glucose concentrations [20]. Figure 4A shows a sample ^13^C NMR spectrum of a perfused liver presented with 20 mM of [1-^13^C] glucose for approximately 50 min, and Figure 4B shows the time course of the [1-^13^C] + [6-^13^C] glycogen NMR signals from a single liver over time for different perfusate concentrations of [1-^13^C] glucose. As can easily be seen, the rate of glycogen synthesis (i.e., the slope of the line in Figure 4B) increases with increasing [1-^13^C] glucose concentration, thus recapitulating the known glucose dependence of hepatic glycogen synthesis in vivo. 

#### 3.2.1. Assay Development for Glucokinase Activators (GKA)

**Figure 5:**
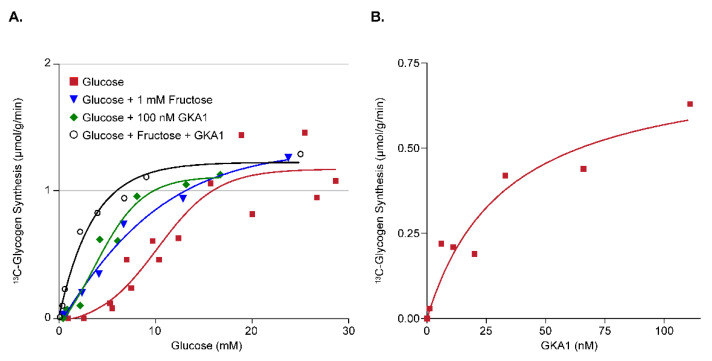
(**A**) The relationship between perfusate [1-^13^C] glucose concentration and ^13^C-glycogen synthesis for untreated livers (red), and livers perfused with 1 mM of fructose (blue), 100 nM of GKA1 (green), or 1 mM of fructose + 100 nM of GKA1 (black). (**B**) The titration of GKA1 at a perfusate [1-^13^C] glucose concentration of 5 mM.

As the liver has a large capacity for glucose uptake and glycogen storage, activation of the enzyme *glucokinase* is a potential strategy for lowering circulating glucose levels via removal of glucose from the blood and storage/disposal by the liver. A well-known indirect activator of *glucokinase* is the sugar fructose, whose phosphorylated metabolite fructose-1-phosphate liberates *glucokinase* from its regulatory protein (GKRP), increasing the amount of *glucokinase* available for glucose phosphorylation [21]. Shown in Figure 5A are plots of the relationship between perfusate [1-^13^C] glucose concentration and ^13^C-glycogen synthesis for untreated livers (red symbols), livers treated with 1 mM of fructose (blue symbols), livers treated with 100 nM of a novel small molecule GKA compound GKA1 (green symbols), and livers treated with 1 mM of fructose plus 100 nM GKA1 (black symbols). This demonstrates that activation of *glucokinase* with fructose and GKA1 increases hepatic glycogen synthesis (via decreased EC_50_) over the physiological range of plasma glucose values and validates this as a viable approach for assessing novel GKA compounds. Shown in Figure 5B is the dose titration of GKA1 at a fixed perfusate [1-^13^C] glucose concentration of 5 mM. This shows that the EC_50_ for GKA1 is approximately 40 nM, which can be used as a guide for plasma levels to target with in vivo dosing. Since multiple concentrations of a novel GKA can be tested in a single liver (similar to the multiple [1-^13^C] glucose concentrations shown in Figure 4B), this approach is a very efficient way of evaluating novel GKAs. Finally, the rates of maximal hepatic glycogen synthesis in the perfused liver (~1 μmol/g/min) are consistent with those observed in vivo in dogs [22] and humans [23] when corrected for typical liver weight to body weight ratios. 

#### 3.2.2. Assay Development for Insulin Analogs and Insulin Receptor Agonists

**Figure 6:**
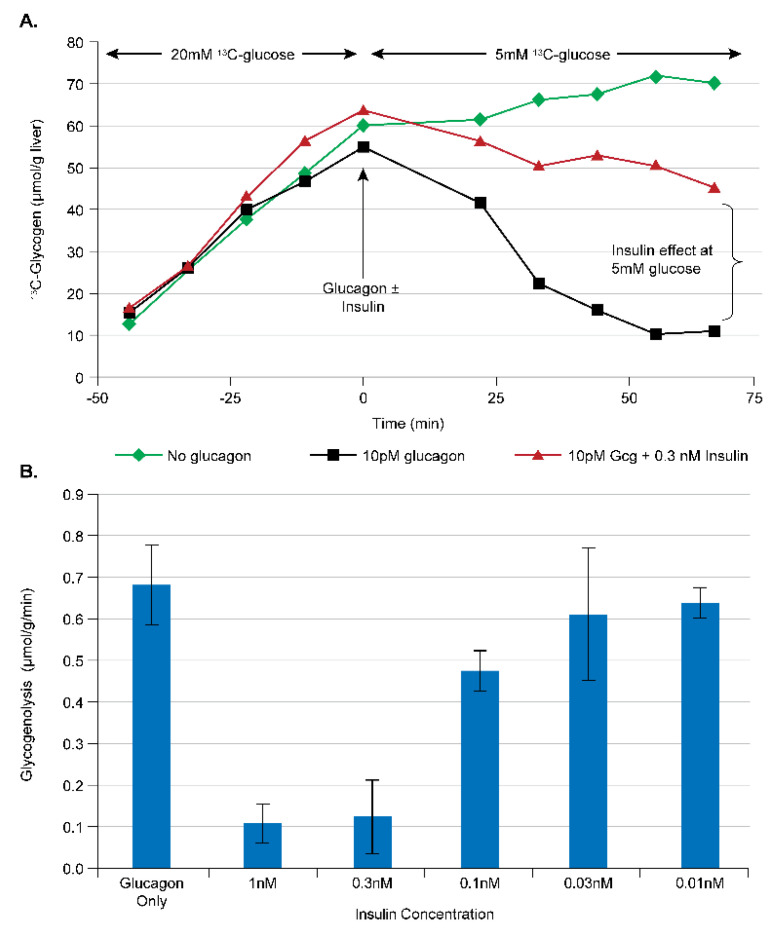
Development of a perfused liver assay of insulin action in C57/Bl6 mouse livers. (**A**) The time course of ^13^C-glycogen for three sample livers subjected to two periods of different glucose concentrations (20 mM for T < 0 min, and 5 mM for T > 0 min) with no intervention (green), 10 pM of glucagon (black), and 10 pM of glucagon + insulin (red). The rate of glycogenolysis after T = 0 min is used to assess the effect of insulin on the liver. (**B**) The dose–response relationship of insulin in perfused liver. Reprinted/adapted from reference [11].

Easily the oldest treatment for diabetes is the administration of exogenous insulin. Due to the risk of hypoglycemia with insulin treatment, however, many diabetic patients are likely undertreated. This has led to the design of insulin analogs, partial agonists, and tissue-specific agonists of the insulin receptor. Assessing the direct effect of insulin (or modulators of the insulin receptor) on the liver in vivo can be challenging, due to simultaneous strong effects of insulin on muscle and adipose tissue glucose/lipid metabolism, and due to the fact that the acute effect of insulin on the liver is primarily on glycogenolysis [16], which is difficult to measure in vivo. The perfused liver again represents a simpler, but still physiologically relevant, system which can assess the effects of insulin or related analogs. Using different concentrations of [1-^13^C] glucose, the rate of ^13^C-glycogen synthesis can be modulated (Section 3.2.1), and, by adding glucagon to the perfusate, glycogenolysis can be stimulated. These effects can then be used to develop an assay of direct hepatic insulin effect as shown in Figure 6. Here, an initial period of 20 mM of perfusate [1-^13^C] glucose is used to stimulate maximal ^13^C-glycogen synthesis and produce a ^13^C NMR-observable pool of glycogen. The perfusate [1-^13^C] glucose concentration is then switched to 5 mM and the liver is treated with (1) vehicle; (2) 10 pM of glucagon; or (3) 10 pM of glucagon + insulin at varying concentrations, during which the rate of glycogenolysis is calculated (Figure 6A). As can be seen from Figure 6B, insulin has an approximate EC_50_ of 200 pM and a steep dose titration with its entire effect being manifest over the 30–300 pM range. This overall dose–response relationship is consistent with what is known about the control of hepatic glycogen metabolism by insulin in vivo [16]. Furthermore, the observation of a direct effect of insulin at these concentrations is novel, and is in contrast to other reports using in vitro systems where the effect of insulin on hepatocytes or other liver-derived cell lines was observed only at supra-physiological concentrations [24,25], or sometimes not at all [26]. 

## 4. Gluconeogenesis

### 4.1. Gluconeogeneiss from [2-^13^C] Pyrzuvate

**Figure 7:**
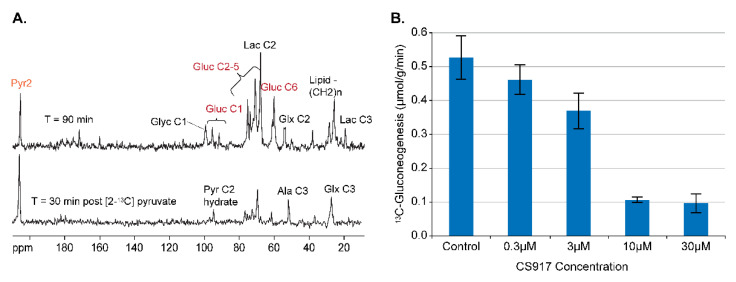
Development of an assay of GNG in db/db mouse liver. (**A**) ^13^C NMR spectra 30 and 90 min after the administration of 10 mM [2-^13^C] pyruvate, illustrating the metabolism of [2-^13^C] pyruvate to ^13^C-glucose, along with other metabolites. (**B**) The dose titration of the F-1,6-BPase inhibitor CS-917, a known inhibitor of GNG.

In addition to storing glucose as glycogen, the liver also has the largest capacity of any organ to produce glucose from substrates at the level of the TCA cycle and export into the circulation (gluconeogenesis). Modulation of hepatic gluconeogenesis (GNG) represents an additional pathway to target when developing glucose-lowering diabetic therapies, and reduction in hepatic GNG was long hypothesized to be one of the mechanisms by which Metformin exerted its therapeutic effects. As can be seen in Figure 7A, [2-^13^C] pyruvate is also a suitable substrate for developing assays of GNG as it is rapidly converted to different combinations of [1-,2-,5-, and 6-^13^C] glucose. 

### 4.2. Assay Development for Inhibitors of GNG

The tool compound CS-917 is an inhibitor of fructose-1,6-bisphosphatase, which catalyzes the conversion of fructose-1,6-bisphosphate to fructose-6-phosphate, a key enzymatic step in the GNG pathway [27]. The addition of CS-917 to the perfusate resulted in a dose-dependent reduction in GNG, as shown in Figure 7B. In addition, livers perfused with CS-917 showed an additional ^13^C NMR signal from ^13^C-fructose 1,6-bisphosphate, confirming the MOA for CS-917 (unpublished observations).

### 4.3. Assay Development for Inhibitors of Malic Enzyme

**Figure 8:**
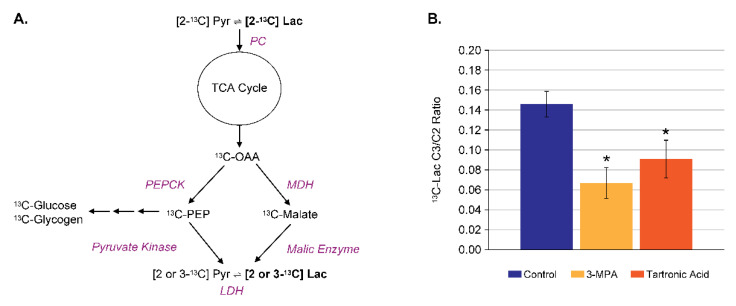
The assessment of pyruvate cycling in perfused liver. (**A**) Schematic of the main pyruvate cycling pathways in liver, where [2-^13^C] pyruvate can be recycled to [3-^13^C] pyruvate via the combination of (1) PEPCK and pyruvate kinase, or (2) malate dehydrogenase and ME1. (**B**) The effect of the PEPCK inhibitor (3-MPA) and the ME1 inhibitor (tartronic acid) to reduce the ratio of [3-^13^C] lactate to [2-^13^C] lactate, a biomarker of pyruvate cycling. * *P* < 0.05 compared to Control.

In addition to the incorporation into glucose and glycogen, [2-^13^C] pyruvate is also rapidly converted to [2-^13^C] lactate via lactate dehydrogenase and [3-^13^C] lactate after subsequent turns of the TCA cycle (Figure 7A, 69 ppm and 21 ppm). The recycling of pyruvate via the TCA cycle is termed pyruvate cycling and is used to generate reducing equivalents in the form of NADPH which is used in lipid biosynthesis. Cytosolic malic enzyme (ME1) is involved in this overall process, as shown in Figure 8A, and there is evidence of a causal linkage between ME1 and obesity in rodents [28]. Thus, pyruvate cycling may be a good biomarker of ME1 activity. A perfused liver-based assay of pyruvate cycling was developed using the ratio of [3-^13^C] lactate to [2-^13^C] lactate, measured after perfusion with 5 mM of [2-^13^C] pyruvate for 90 min [29]. In this case, the perfusate was not recirculated to keep the level of [2-^13^C] pyruvate constant and to prevent the re-metabolism of ^13^C-lactate which would contaminate the assay readout. Overall, this is analogous to the approach of Petersen et al. in vivo [30]. Figure 8B shows the effect of the ME1 inhibitor tartronic acid [31] at 10 mM in the perfusate on this pyruvate cycling ratio as well as the effect of the phospho-enol pyruvate carboxykinase (PEPCK) inhibitor 3-mercaptopicolinic acid (3-MPA) at 300 μM which blocks the other pathway of pyruvate cycling, as shown in Figure 8A.

### 4.4. Application to GPR40 Agonists

**Figure 9:**
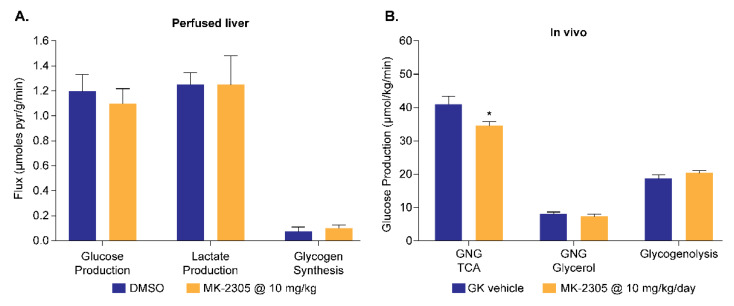
Using the perfused liver assay of GNG as part of an effort to elucidate the glucose lowering MOA of the GPR40 agonist MK-2305. (**A**) The lack of a direct effect of 10 μM of MK-2305 on hepatic GNG in perfused db/db livers. (**B**) The in vivo effect of MK-2305 dosed at 10 mg/kg of PO to reduce GNG in GK rats in vivo using the ^2^H_2_O approach. These results demonstrate that MK-2305 treatment indirectly reduces GNG. * *P* < 0.05 compared to GK vehicle. Adapted from reference [34].

The GPR40 receptor is a fatty acid receptor whose activation results in glucose lowering and enhanced insulin release in vivo. GPR40 agonists have been shown to provide a therapeutic benefit in preclinical models of diabetes [32] and also in clinical trials [33]. The perfused liver assay of GNG was employed as part of an effort to elucidate the therapeutic MOA of the GPR40 agonist MK-2305 [34]. GNG was measured in db/db mouse livers perfused with [2-^13^C] pyruvate which were treated acutely with 10 μM of MK-2305 or vehicle (0.1% DMSO). No direct effects of MK-2305 on GNG, lactate production, or glycogen synthesis were observed (Figure 9A). Conversely, separate studies using the ^2^H_2_O approach to measure the sources of glucose production in vivo [35] in Goto-Kakazaki rats showed that PO treatment with 10 mg/kg/day of MK-2305 decreased endogenous glucose production, primarily via the reduction in GNG (Figure 9B). These results suggested that GPR40 activation with MK-2305 treatment indirectly reduces GNG to improve glucose homeostasis, presumably via enhanced insulin secretion.

## 5. Interaction of Glucose and Lipid Metabolism

### 5.1. Metabolism of [1-^13^C] Oleate

The interaction of glucose and lipid metabolism was observed over 50 years ago by Randle and colleagues and was initially termed the ‘Glucose–Fatty Acid Cycle’ [36]. While the exact mechanisms underlying this interaction have been extensively debated, many subsequent studies have demonstrated that alterations in lipid levels in metabolically active tissues have a profound effect on glucose metabolism [37,38,39]. Similarly, studies on pharmacological agents that modulate fatty acid oxidation (FAO) have been shown to impact circulating glucose levels and responses to glucose challenges [40,41]. 

**Figure 10:**
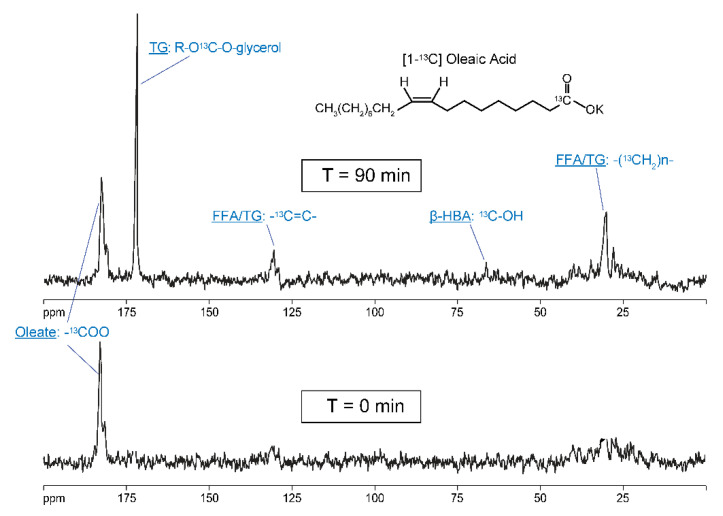
Development of a perfused liver assay of lipid metabolism. Shown are spectra acquired as early as possible after liver excision (T = 0 min), and after 90 min of metabolizing [1-^13^C] oleate. The NMR signals in the top spectrum demonstrate the ability to measure the synthesis of triglycerides, fatty acid oxidation, as well as recycling of lipid in real time.

The perfused liver platform is also well suited for the study of hepatic lipid metabolism, a convenient substrate for which is [1-^13^C] oleate. The only modification to the perfused liver procedure when using long chain fatty acids as substrates is that bovine serum albumin (BSA) must be added to the perfusate and that fatty acids need to be pre-formulated with the perfusate. Hence, natural abundance background spectra prior to substrate addition cannot be collected. As can be seen in Figure 10, [1-^13^C] oleate is primarily metabolized to ^13^C-labeled triglycerides. The metabolism to ^13^C-β-hydroxy-butyrate (β-HBA) via FAO is also significant; however, the NMR signal from this ketone body appears artificially low for the following two reasons: (1) it is exported from the liver into the perfusate and thus largely out of the detection volume of the NMR probe; and (2) only the first round of β-oxidation generates ^13^C-labeled ketone bodies from the ^13^C label in the 1 position of oleate. As a result, it is often more convenient to assay the perfusate for ^13^C-β-HBA via traditional biochemical methods and combine this with solution NMR analysis of the ^13^C enrichment in β-HBA. Alternatively, a substrate, such as [2,4,6,…,16-^13^C] palmitate, can be used to generate ^13^C-βHBA from each round of FAO and thus boost its NMR signal [42]; however, this has to be balanced against the high cost of this substrate. Furthermore, note that a minor portion of the oxidized [1-^13^C] oleate is also used as a substrate for *de novo* lipogenesis and is recycled back into lipids, though this is generally only observed for perfusate levels of [1-^13^C] oleate > 1 mM and in lipogenic mouse models (e.g., db/db, ob/ob, high fructose fed).

### 5.2. Combined Metabolism of [1-^13^C] Oleate and [1-^13^C] Glucose 

**Figure 11:**
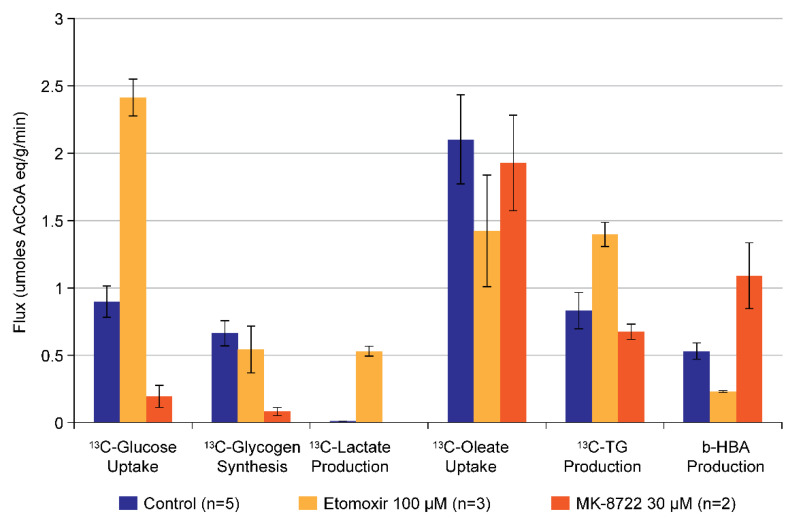
Using the perfused liver to investigate the pharmacological modulation of the interaction of glucose and lipid metabolism. Shown are fluxes through the detectable metabolic pathways from [1-^13^C] glucose (left three quantities) and [1-^13^C] oleate (right three quantities), as well as the effects of the CPT1 inhibitor etomoxir and the AMPK activator MK-8722. Note the striking difference in the pattern of effects from each compound.

While [1-^13^C] oleate can be used on its own in the perfused liver to gain insight into fatty acid metabolism, it can also be co-administered to the liver along with [1-^13^C] glucose to gain a more wholistic understanding of the effects of novel compounds on *combined* glucose and lipid metabolism. This is because of the following two reasons: (1) following oxidation of each substrate, the ^13^C label from [1-^13^C] glucose and [1-^13^C] oleate appears in different locations (position 2 and 1, respectively) in acetyl-CoA prior to entry into the TCA cycle; and (2) de novo lipogenesis from [1-^13^C] glucose is very low during the time scale of a typical perfused liver study (60–90 min). This combined substrate approach was used to understand the mechanism of action of two different pharmacological agents, the well-known carnitine palmitoyl transferase (CPT1) inhibitor, Etomoxir, and the AMP-activated protein kinase (AMPK) activator, MK-8722 [43]. The major metabolic fluxes related to each of these substrates are shown in Figure 11, where the individual fluxes are presented in terms of acetyl-CoA equivalents to make the relative comparison more straightforward (e.g., 1 glucose = 2 Ac-CoA, 1 Oleate = 9 Ac-CoA, etc.). As can be observed, acute CPT1 inhibition increases hepatic glucose uptake, increases glycolysis (es evidenced by increased lactate production), increases triglyceride synthesis, and decreases ^13^C-βHBA production, consistent with the well-known role of CPT1 in FAO. Conversely, the AMPK activator MK-8722 decreases hepatic glucose uptake, decreases glycogen synthesis, and increases ^13^C-βHBA production, consistent with the known effects of AMPK activation on glycogen synthase and acetyl-CoA carboxylase 2, respectively. Interestingly, both compounds have been shown to improve diabetic symptoms in preclinical models [40,44], though the data in Figure 11 suggests this is through very different mechanisms. 

## 6. Summary and Other Applications

The perfused liver platform represents an efficient means by which to evaluate the effects of novel compounds on hepatic metabolism because it captures the essential features of glucose metabolism and its known hormonal and pharmacological controls. In particular, using the perfused liver, we were able to demonstrate the following: (1) the ability to synthesize glucose from three carbon precursors (i.e., GNG) at rates consistent with those observed in vivo; (2) the inherent glucose-dependent nature of hepatic glucose uptake and glycogen synthesis; (3) maximal rates of hepatic glycogen synthesis which are consistent with published rates from in vivo studies; (4) the ability of glucagon to rapidly stimulate glycogen breakdown at physiologically relevant concentrations; and (5) the ability of insulin to modulate the glucagon-induced glycogen breakdown at physiologically relevant concentrations.

While this review focused on applications to glucose metabolism, other ^13^C labeled substrates can be used to selectively probe different metabolic pathways. For example, ^13^C-fructose is readily metabolized by the liver and can be used to investigate the effects of modulating the enzyme ketohexokinase, which is of current interest as dietary fructose consumption increases. ^13^C-glycerol is a potentially useful substrate for studying the inhibition of gluconeogenesis for targets above the triose phosphate isomerase step in the gluconeogenic pathway as it yields simpler spectra than a gluconeogenic substrate such as ^13^C-pyruvate. For select targets related to the FAO pathway, a short chain fatty acid, such as ^13^C-octanoate, may be a more optimal choice than ^13^C-oleate as it bypasses the FAO rate-controlling step of CPT1 and results in increased NMR signal from ^13^C-βHBA, which may improve assay development. 

In summary, with appropriately chosen ^13^C labelled substrates, we were able to develop perfused liver assays to assess the effects of novel compounds and targets on biochemical pathways related to hepatic glucose metabolism. We were able to show acute effects of pharmacological tool compounds in the perfused liver consistent with their reported mechanisms of action. These tool compounds served to validate perfused liver assays for the assessment of novel molecules and mechanisms, which could then be used as a simpler and more economical approach to facilitate drug development.
